# American Dental Association—Twenty-Fifth Annual Meeting

**Published:** 1885-10

**Authors:** 


### American Dental Association—Twenty-Fifth Annual Meeting.

REPORTED BY MRS. M. W. J.

The twenty-fifth annual meeting of the American Dental Association was held at Minneapolis, commencing August 4, 1885. This was the largest and most successful meeting ever held by this Association. The attendance was unusually large (four hundred); the number of new members admitted (three hundred and four) unprecedented. The papers read were marked by deep and thorough scientific research and investigation, and the discussions were earnest and animated.

Delegates were present from all the most prominent state, district, and local societies, the dental colleges, and the dental departments of several universities. From New England to New Orleans, from the District of Columbia to the far North, South, East, and West, were well represented.

The officers, committees, and sections were as follows:

OFFICERS.

President, J. N. Crouse, Chicago, Ill.
First Vice-President, M. W. Foster, Baltimore, Md.
Second Vice-President, C. F. Rich, Saratoga, N. Y.
Recording Secretary, Geo. H. Cushing, Chicago, Ill.
Corresponding Secretary, A. W. Harlan, Chicago, Ill.
Treasurer, Geo. W. Keely, Oxford, O.

EXECUTIVE COMMITTEE.

C. N. Peirce, Chairman; A. M. Dudley, Secretary.

FIRST DIVISION—COMMITTEE OF ARRANGEMENTS.

J. N. Crouse, Geo. L. Field, W. N. Morrison.

SECOND DIVISION—ON CREDENTIALS, ETHICS, AND AUDITING ACCOUNTS.

A. M. Dudley, L. G. Perry, A. O. Hunt.

THIRD DIVISION—ON VOLUNTARY ESSAYS.

C. N. Peirce, L. D. Shepard, Geo. J. Friedrichs.

LOCAL COMMITTEE OF ARRANGEMENTS.

A. T. Smith, of Minneapolis, F. H. Gardner, of Chicago, and, H. J. McKellops, of St. Louis.

SECTIONS.

*Section VII. Physiology and Etiology.*—H. A. Smith, Chairman; N. W. Kingsley, Secretary.

*Section I. Prosthetic Dentistry, Chemistry, and Metallurgy.*—John Allen, Chairman; W. H. Trueman, Secretary.

*Section II. Dental Education.*—A. O. Hunt, Chairman.

*Section III. Dental Literature and Nomenclature.*—J. Taft, Chairman.

*Section IV. Operative Dentistry.*—L. D. Shepard, Chairman; V. H. Jackson, Secretary.

*Section V. Anatomy, Histology, and Microscopy.*—C. F. W. Böedecker, Chairman; Frank Abbott, Secretary.

*Section VI. Pathology, Therapeutics, and Materia Medica.*—A. W. Harlan, Chairman; F. M. Odell, Secretary.

The meeting was called to order at 10 A.M., with the President, Dr. J. N. Crouse, of Chicago, in the chair. The first morning session was devoted to the work of organization, the usual routine business, reports of committees, etc.

Dr. E. Parmly Brown, Chairman of Committee, asked that the committee appointed at the last meeting, for securing larger attendance of lapsed members, be discharged, further efforts being unnecessary by reason of the present large attendance. Information was given that old members who had lost their membership by non-attendance and non-payment of dues would be reinstated on payment of two years' past dues. Committee discharged. Drs. Smith, of Minneapolis, Gardner, of Chicago, and Field, of Detroit, were appointed a special committee to make arrangements for the half day devoted to clinics.

The Committee on Publication announced that, through arrangements renewed with the S. S. White Manufacturing Co., their work had been done almost without cost to the Association. That the thanks of the committee were tendered to Mr. Hill for valuable assistance rendered. That the business minutes of the Association from its very organization in 1856, had been compiled from the original MSS. and published for the benefit of members of the Association. On motion of Dr. H. A. Smith, of Cincinnati, the transactions were ordered bound in more substantial manner, suitable for library purposes.

The Secretary, Dr. Geo. H. Cushing, announced the death, during the past year, of Dr. J. G. Ambler, of New York, and Dr. Isaiah Forbes, of St. Louis. Drs. Taft, Spalding of St. Louis, and Atkinson were appointed a Committee on Necrology.

Dr. T. T. Moore, of South Carolinia gave notice of a motion to amend Article 4 of the Constitution by striking out "first Tuesday" in August, and substituting "fourth Tuesday." This lays over till next year.

EVENING SESSION.

Called to order at 8 P. M. The President in the chair.

The Committee on Voluntary Essays announced three papers which they recommended be read.

One from Dr. W. H. Atkinson, of New York, on "Pyorrhea Alveolaris;" one from Dr. J. A. Robinson, of Jackson, Mich., entitled, "Painless Operations," and one from Dr. L. C. Ingersoll, Keokuk, Iowa, on "The Alveolar Dental Membrane." Also a paper by Dr. E. Parmly Brown, entitled, "Dentistry: Its Past; Its Encouraging Present; Its Brilliant Future," which was left over from last year from lack of time for reading.

On motion, the report was accepted.

The Treasurer reported as follows:

| | |
|---|---|
| Balance on hand | $1,823.95 |
| Received since last meeting | 400.00 |
| | $2,223.95 |
| Expended as per Vouchers | 275.00 |
| Balance on hand | $1,948.95 |

The report was referred to the Auditing committee.

The President stated that his time had been so fully occupied by his duties as Chairman of the Committee of Arrangements, that he had not been able to prepare the customary formal annual address. He hoped the results of his labors on the Committee would prove more acceptable than a speech.

He would only ask the members to bear in mind that they did not come together merely to elect officers and have a good time, but that they had legitimate work to do. He would make two recommendations: first, that more work be done by the various sections between the annual meetings, thus facilitating work during the Session; and, second, that the Association make appropriations for legitimate scientific work, and original investigation in the sections; that this would be the most appropriate disposition of the annual income.

This proposition was received with hearty approbation, and a committee appointed to consider the subject.

Section VII, Physiology and Etiology, reported through Dr. A. H. Thompson, Topeka, Kansas, sec. *pro tem.*, a paper by Dr. W. C. Barrett, Buffalo, N. Y., entitled, The Earthy Phosphates. Also a recommendation that the Association appropriate the sum of $200 for the prosecution of scientific investigation, by Section VII.

Brief summary of Dr. Barrett's paper: The subject of the administration of the earthy phosphates to pregnant woman had given rise to many plausible theories and elaborate speeches on the growing demands of the fœtus, the system of the mother being robbed, her teeth consequently becoming soft and decaying, the lime-salts of her teeth going to form those of the child, etc., etc. Incidents were cited of the wonderful results of the administration of lime-salts to the mother to prevent all this.

Failures to obtain the desired results were not reported, or, if mentioned, were attributed to lack of faithfulness in following instructions. But even where the earthy phosphates are most faithfully administered, where dystrophic conditions exist there will be found quite as many defective teeth as where nothing of the kind is attempted. He said he had early experimented in this direction, and had considered his first case a marvelous success. The lady's first child had had deplorable teeth. In the case of the second child, as the seeming result of the administration of lime-salts, the teeth were remarkably fine, and were erupted without any febrile disturbance, etc.

In the next case, however, the results were quite the contrary. It was the fifth child; the teeth of the first four were fairly good, but those of the mother abnormally soft. He administered the lime-salts with most confident expectations, but the fifth child had the only really bad denture in the family.

The result of the first case was never repeated, and long-continued experience had finally led him to abandon both the theory and the practice. The study of physiology and nutrition forced him to conclude that it was useless to administer the phosphates. The facts also were all against it.

A few years ago the dental journals were filled with denunciations of fine flour. It was demonstrated to the satisfaction of many that decay of the teeth was due to a lack of phosphates. Later computations, however, show that fine flour alone furnishes the system an excess of lime-salts, and that they are always eliminated from the system during gestation, no matter what the diet may be. Nutritive changes in the teeth are very slow. Dystrophy may effect them, but progressive changes are not manifest. There are physiological reasons why the phosphates are not nutritive; that the theory is erroneous. No order or class of animals can derive nourishment directly from mineral elements; they cannot digest inorganic matter. The mineral elements must be taken up by the vegetable kingdom and organized; they then become food elements for graminivorous animals. The carnivora cannot take the mineral elements even when organized by the vegetable kingdom, but they must be organized a second time by the graminivora before they become food for the carnivora. Man is omnivorous and uses food elements indifferently whether once or twice organized.

The earthy phosphates, when introduced directly into the system, will be eliminated unchanged, or they will act as an irritant and create disturbance; they are either useless or mischievous. It is true that the toothless class swallow inorganic matter, but it is for a mechanical purpose, serving as teeth, and not for food.

There are some proximate principles in the human body which are inorganic, as for instance iron, but which are not assimilated directly. The calcium of the bones is not assimilated directly from the mineral kingdom, but is elaborated within the organism and appropriated. We cannot build the walls of our physical bodies by swallowing bricks and mortar. The carbonate of lime cannot be assimilated from oyster shells, but from pabulum.

If this were not so it would not be possible for the human race to live under such different conditions as exist in the Arctic regions, where there is no vegetable food, and at the equator, where animal food is injurious; but a small portion of the earth would be habitable if it were necessary to have the elements combined in exact proportions. Animal life is sustained by organic matter, while inorganic matter acts as an actual irritant. In abnormal conditions they have their value as remedial agents, but not as food. When necessary to correct dystrophic conditions, inorganic matter may produce such peristaltic action as is required, but it is foreign to the organism and has no part in nutrition ; it is not built up into tissues. If they have no decided medicinal properties there is no excuse for dispensing such inert compounds, which are always eliminated unchanged.

The Chair. The paper and subject are now open to discussion.

Dr. C. W. Spalding, St. Louis, said that he would not attempt to refute the general position taken in the paper, but that there were some points difficult to reconcile. As a general rule, the inorganic elements are not appropriated directly, as food, but having passed through the vegetable tissues are used advantageously. As medicine they certainly have their value. There is one inorganic substance that is largely and generally used that would seem to constitute an exception to the rule, viz: sodium chloride, common salt, which is a mineral substance that is used habitually by both man and beast. It is true that it is eliminated unaltered, but it has never been shown that *all* that is taken is thus eliminated; I am not aware that the quantity eliminated has ever been proved to be equal to the quantity that is taken in. May not a portion of it undergo decomposition within the system, and the elements be incorporated into the tissues? He said that he did not assert this to be the case, but would raise the question. Wild animals seek for salt so habitually that hunters know where to look for them from this fact. With domestic animals this might be a perverted taste, a habit acquired in domesticity, but this could not be the case with wild animals. It must be a necessity of the animal economy. Is this salt decomposed and incorporated, or is it only used in a remedial or economic way?

Dr. Pierce, Philadelphia: The paper sets forth ideas seemingly antagonistic to the laws governing nutrition. The idea is thrown out that man is the only animal requiring therapeutic remedial agents. Now we know that some animals eat clay; the dog and the cat, which are carnivorous, eat grass, in certain conditions, as a remedy. It was said that the animal system was not competent to appropriate inorganic material, but every one knows that if chickens are deprived of lime their eggs will have no shells, indicating some deficiency in the system. We mystify ourselves by talking of organic and inorganic elements. When lime has been taken up by vegetation it is still lime and no less inorganic.

The inorganic salts are taken indirectly through the vegetable kingdom, but they are none the less inorganic because of passing through the vegetable kingdom. Lime is taken up by vegetation and the soil exhausted of this element, more lime must be given to the soil to supply future crops. In certain cases we can benefit patients and aid assimilation by the administration of lime-salts.

Stomach fermentation is relieved by lime-water; it thus aids nutrition. The essayist got "the cart before the horse" when he said the animal was made for a certain diet. Diet makes the animal; the food-habit gives form and character to the organism. Take an animal of any group—carnivorous, graminivorous, herbivorous—change the diet, accustom it to a new diet, and the whole system is changed. The different tooth-forms are the result of difference in diet. There is lime on the earth, in the air, in the water. Similar food-habit has made similar tooth structure, the same teeth because of the same food-habit. Dogs are carnivorous; their teeth are what they are because of their food-habit. The Polar bear is truly carnivorous; the bear of the mountains has a grinding molar from different diet. Food and function must go hand in hand. In the human family there is a large class of children who always have a cup of fluid at hand to wash down each mouthful of food. Their teeth are soft, and soon break down ; the tooth function has been delegated to the stomach. Their food contained lime enough, but the teeth were not exercised; hence the universal pathological condition called caries.

Dr. Atkinson said he wished to congratulate Dr. Barrett on his growth ; he was formerly the man *par excellence* to uphold the theory he now cries down.

As he said, it is truly an easy thing to find testimony in favor of our theories, and to pass by all that is the contrary. " One man's meat may be another man's poison."

The same article may be food, medicine or poison according to conditions, acting by reason of awakening latent energies roused by deficiencies in the system.

The classification of organic and inorganic is simply a bedevilment of words. As the latest upheavals of mountains are the highest, so with words. We must modify our views ; tear down false gods ; be sure we are right, then go ahead. The subject is very complicated. We must have the ability to comprehend how pabulum is produced, or we may hammer away in vain till the crack of doom. There must be correct, serial, orderly arrangement of statement. A multiplicity of assumption is held of equal value with facts.

What is the nature of the demand we call hunger ? It is difficult to comprehend. It is an awakening of latent energies. We are not hungry in the stomach, but where the pabulum has been burnt out. Some one said that salt passes out without being changed ; he had better revise his animal chemistry.

All the oxygen that has passed within the grasp of the lungs is as dead as a door-nail, although only four per cent. has been appropriated ; and so with salt. Of some things we can simply say, " We dont know." Another says the food habit forms the teeth ; that the food-habit is the mother of tooth-form ; rather the ghost of a tooth ! It is assumed that matter does all things.

Scientists, so-called, materialists, are the wisest in assumption. An assumption is made the basis of a statement. An opposite assumption cancels the first, and so on. The awakening of latent energies in so-called inorganic matters renders them capable of acting. Physicists are not willing to admit that there is any power in the atom.

Dr. C. W. Spalding. Does Dr. Atkinson say that common salt does not pass through the system unchanged?

Dr. Atkinson. I do. There must be a change in the salt, as there is in the oxygen. Oxygen that has passed through the lungs is nil for human inhalation, though only four per cent. has been appropriated. Salt has been similarly changed, though not to the same extent with oxygen.

Dr. Spalding to Dr. A. Shall we report you as saying that salt does not reappear unchanged?

Dr. Atkinson. I don't care; report what you please.

Dr. Frank Abbott. Said he had gone through the experiments with the lime phosphates, with the same results. In no particular instance had there been marked improvement. All but one case had failed. He had obtained no better results with treatment than without. In certain experiments with rabbits—two having broken legs—the one treated with lacto-phosphate of lime healed more readily than the one not so treated. Lacto-phosphate of lime was given to the world as the much needed remedy for the teeth. It succeeded with the rabbit's leg but fails with the human teeth. Has not yet given up all hope, but is still looking for the right thing. The form in which it will be assimilated has not been found; perhaps because the phosphate we buy is not the pure phosphate; it is dissolved in lactic-acid; lime three parts, phosphorus one, is ordinary formula. Except in the form of food we fail, but every person takes in his ordinary food sufficient lime-phosphate for every purpose. Ladies in certain circumstances, however, do not retain enough food; so much is rejected, from constant nausea, that they do not obtain from their food the quantity required for nutrition. This is the cause of the trouble with their teeth. One speaker said that certain elements were taken to stimulate latent energies that had previously been depressed. We see the teeth and know they are defective; probably other organs are also defective, but we cannot see them as we do the teeth; hence in the latter organs only we recognize the deficiency.

Dr. Pierce, Philadelphia. I said the food-habit was the mother of tooth-forms. If we go into a museum with a pair of dividers and examine the lower jaw of any animal, placing an arm of the dividers on the condyle and decribing the valleys in the teeth; in another class of animal, placing the arm half-way between the condyles, we shall find that the movement of the jaw has been the mother of the shapes of the crowns of the teeth.

One class, the carnivora, has dentine in the centre, enamel on the crown, and cementum on the roots; another class has the enamel on the labial surface and cementum on the palatine surface, and the incisors always sharp. The jaw of the rodent has a lateral motion; the enamel is not on the crown, but side by side with the dentine; the movement on the jaw has given the tooth-form.

Dr. L. C. Ingersoll, Keokuk, Iowa, asked Dr. Pierce if he ment to say that tooth-form and functions were governed by food-habits.

Dr. Pierce. The necessity for anything provides its presence. In the absence of function there is absence of nutrition. Function causes nutritition; if nutrition is delegated to another part, function follows it.

Dr. Ingersoll considered it a question whether the formation of the stomach is the result of the formation of the teeth. Is cud-chewing the mother of the double-stomach?

Dr. Pierce. There is much obscurity in many forms of development, but I still adhere to the principle that function is the mother of organization. The food-habit modifies the alimentary canal, as is seen by the comparative anatomy of herbivorous and the carnivorous animals.

Dr. C. W. Spalding. If the food-habit controls the conformation and measurement of the jaws, I would ask when the habit originated? What governed the conformation of the first animal? If the developement of stomach and teeth is contemporaneous—if the stomach only develops when the teeth indicate the need for solid food—how about the stomach when the teeth fail to appear? If a child of two years old has erupted no teeth, has it no stomach? There is a man in Pennsylvania who is forty years old, who has never had any teeth; he is in vigorous health, though he has no sweat-glands and has no hair. Has he no stomach?

Dr. Sitherwood, Bloomington, Ill. The results of my reading and observation agree with the position taken in the paper. There are seeming exceptions, but the rule holds good. Too much confidence is placed in feeding mineral substances. I have always been the friend of children. I left at home a sick child, ten months old. Our physician has had twenty-five years' more experience than I have, but when I left home I gave orders that the child should have no more of the patent "infant's foods;" no mineral elements, even as medicine--only pure cow's milk and vegetable stimulants. Since my arrival I have had two dispatches and the child is improving rapidly. The necessity of lime for egg-shells, and for salt in the human system forms two apparent exceptions to the rule; but we do not fully understand these things yet; I have no faith in administering mineral substances as elements of nutritition.

Dr. King, Lincoln, Nebraska. We are assuming some things that we cannot fully sustain. Dr. Atkinson assumes that when we are hungry, the hunger is not in the stomach, but in the part that needs to be supplied. My experience is that as soon as the stomach is filled, hunger is satisfied, though the parts that need the supplies are not yet reached. Marked results from the administration of the lime-salts cannot be expected immediately.

Dr. Atkinson. When what I say is impugned I want either to prove it or be corrected. The proof that hunger is not in the stomach is seen in the common practice of the Indian who, when he is hungry and food is not at hand, will, by tightening his belt, bring the walls of his stomach in closer contact, so as to transmit the blood that contains food elements, and the want is satisfied, though the stomach is not filled.

Dr. Barrett. I wish that the subject presented in the paper had been more closely adhered to. We come here, not to discuss elementary principles, but should come prepared to comprehend fundamental, basal principles. The hunger of the tissues is felt in the stomach. Supply the stomach with pabulum and hunger is satisfied; but it is also satisfied with enemas. Thirst is satisfied by fluids in the stomach, but it is also satisfied just as quickly by injecting the fluid into the veins.

Certain tissues can be fed without the intervention of the stomach. Our nomenclature is deficient. The terms organic and inorganic are unsatisfactory.

Water is inorganic. Because of paucity of words our nomenclature and terminology are very unsatisfactory; but we must use common terms in common discourse. It is the general law (which has its exceptions) that the animal economy, under no circumstances, under trophic conditions, can use, as nutrition, inorganic substances.

The vegetable organizes the inorganic; then the animal can take it. One speaker cited, as a triumphant refutation, that lime was necessary for eggshells. The shell is no portion of the egg *per se;* it simply affords extraneous protection. The lime forms this covering, but it is not essential to the egg.

Egg is egg, ovum is ovum; whether in or out of the body, with or without shell or lime-salt. When incubation takes place outside of the body, lime is required for external protection, but forms no part of the egg itself––

Dr. G. J. Friedrichs, New Orleans. May I ask one question?

Dr. Barrett. I do not like to have my argument interrupted, but go on, if the question is of importance.

Dr. Friedrichs. You stated that the shell was no part of the egg. Now it is stated in works on physiology that the young absorb lime from the shells to form the bones; therefore, if this is true, the shell is essential.

Dr. Barrett. The remark is not worth answering. Animals which are oviparous have eggs without shells. When incubation takes place within the body there is no shell; therefore the shell is not essential to the ovum. Dr. Atkinson has proved that inorganic elements play no part in nutrition—are not built up into tissues. Their part is to hold in solution some other essential elements.

Chloride of sodium is essential to osmosis; it changes the density of certain fluids so that osmosis and exosmosis can be caried on. From flint and steel we get fire, but we cannot strike fire from a roll of butter. I like sharp discussion. From the concussion of flint and steel we may strike the fire of truth. With regard to the average length of the alimentary canal; in the carnivora it is five times the length of the body; in the omnivora, thirty times. This teaches that the higher the organization of pabulum the shorter the digestive tract; or, as a general law, food elements that have been twice organized require a shorter digestive tract For a long time we taught the administration of of earthy phosphates to the mother, for the nutrition of the child. This was through a misconception of the process of digestion and assimilation.

The system will not take these elements from any ready-made source. Nature abhors a ready-made garment. If the tissues need the carbonate or phosphate of lime they will be elaborated within the system, but the system will not take the inorganic carbonate or phosphate of lime and build it up into the system.

Dr. Pierce (by consent) It is well known that the rocks which form the foundation of the city of Paris are made up of infusorial animals. The lime was taken out of the sea by these infusoria; these rocks are miles in extent, and thousands of feet deep. This lime is already organized, having been taken up and assimilated by these microscopic animals.

Dr. Barrett. Do you call the product of the coral polyp, organized?

Dr. Pierce. It is as much organized as the lime that is taken up in grain.

Dr. Barrett. Vegetables do take it up and organize it; animals do not.

On motion adjourned to 9 A. M. Wednesday.

WEDNESDAY AUGUST 5.—MORNING SESSION.

Called to order, the President, J. N. Crouse, in the chair.

Minutes of the preceeding meeting read and approved.

Prof. Pierce of Philadelphia, moved that a committee of three be appointed to take into consideration the suggestions of the President relative to appropriations from the treasury for the advancement of scientific investigations, and to report on the propriety, advisability, and expediency of adopting the suggestions.

The chair appointed Drs. Pierce of Philadelphia, Dr. A. H. Thompson of Topeka, and Dr. E. D. Swain of Chicago.

Dr. Morgan said that he was in favor of the committee, but that he opposed the appropriation.

The discussion under Section VI. was continued.

Dr. Morgan, Nashville, Tenn., said that one speaker had assumed that the peculiar character of development was due to food-habit. The fact was, that development followed type, the type being the result of contending forces, he had got "the cart before the horse." Type forms were modified by food-habit, but were not created by it. The type was also modified by exercise and use.

Whenever a want is expressed in the animal economy, a desire mental or physical, it carries with it a promise of satisfaction.

There is a call for, or a need of, the salts of potassium, or of calcium, and nature has provided the supply everywhere; but he deprecated the idea of introducing the crude inorganic material as nutrition.

The distinction was not very clear between organic and inorganic. A potentiality was conveyed to mineral matter after it was taken into system. The human blood always has iron in it. We may administer the chemical salts of iron and increase the quantity in the system, but we do not know how much vitality it gains after its introduction into the system. He was not prepared to take the broad ground that these elements were never incorporated into the system.

Since there is inorganic matter in all food, no necessity exists for introducing it in the crude form. When the teeth are very inferior, the material necessary to improve them is being thrown off by the system, and incrusted on the teeth. The system has a larger supply than it can appropriate. Ten times or twenty times as much as is needed is eliminated by the system as superflous.

This is true of the perfectly healthy body. The great trouble is in assimilation. It is digested but not appropriated. When the matter of assimilation is regulated there will be no question of administering lime-salts.

Dr. J. R. Patrick, Belleville, Ill., said it had long been recognized as an established point that all matter was included in the two great divisions, organic and inorganic, and that there were three great kingdoms, the mineral, vegetable and animal, and that was the order of creation—the mineral kingdom, the forerunner of the vegetable, and that of the animal which was impossible without it. Some vegetables and animals antedate others, but the consumed must exist before the consumer. The simpler animal forms once created, higher forms become possible. The animal kingdom is not derived exclusively, directly from the vegetable. The lion cannot live on grass like the ox.

Proximate principles are only found in cell formation of the vegetable kingdom, ready for the animal. The chemist has never been able to produce these principles. The alchemist looked forward to the time when he could make a man outside of the regular order—the chemist never.

Mineral matter cannot be appropriated by the animal before being elaborated by the vegetable. Was surprised to hear Dr. Spalding say that salt could be taken into the system and appropriated. Salt, the chloride of sodium, goes into the system, penetrates every part of the body, does its work, and comes out of the system—still chloride of sodium.

Vegetable life is the larval state of the animal. There is an anatomical reason why the tooth cannot be reconstructed. It cannot repair itself except in the cementum. The cementum of any animal is precisely like the bone of the same animal—so exactly the same that the anatomist can classify an animal as readily by the cementum as by a bone. In the cementum, as in a bone, nature forms a matrix for the deposition of new matter. The tooth hardens at the periphery, and is soft in the center—the pulp is the remains of the organic matrix of the tooth. Bone, on the contrary, hardens at the centre, first. One is centripetal in growth; the other is centrifugal. The tooth being hardened at the periphery cannot be reconstructed except in the cementum. If broken below the cementum it may be repaired, but not above the pulp. In old age the whole tooth is solid, but the last dentine deposited is not like the first; the dental energies were exhausted.

Dr. A. H. Thompson, Topeka, said that it depends on the powers of protoplasm. The vegetable elaborates the different elements; the animal appropriates the elements after they have been thus elaborated. We can determine how it is appropriated by the animals, but we cannot understand how it is elaborated by the vegetable.

Animal structures are but modified protoplasm; man differs from the lowest orders in having limbs and muscles to control them; he differs in structure, but is only a larger mass of protoplasm, with rigid materials holding the protoplasm in place. Vegetable protoplasm contains forty elements; animal protoplasm has not been analyzed so exactly. Vegetable protoplasm is almost constant, but animal protoplasm differs greatly; in the teeth it is very dense. The different organs and tissues differ infinitely, and the whole question turns upon the ability to appropriate certain elements. With regard to the administration of mineral matter for building up animal tissues—mineral elements are not food; neither are all vegetable elements food. Morphine, for instance, and many other vegetable products, are medicinal and poisonous, while much of our so-called food is really medicinal.

We cannot direct the elements to any one point with certainty; the organs are selective and take from the pabulum what they most need. In the administration of food we cannot be certain that it will benefit any one organ. Assimilation is the one great point in nutrition; an organ may be very weak and not able to select the proper elements for its own use, or to derive sustenance from the pabulum.

Dr. C. W. Spalding, said that the gentleman from Illinois had misunderstood him; that the point he had endeavored to make clear was that salt was taken into the system by man and beast, and excreted unchanged. And that he had raised the question whether any part was appropriated, as it had not been determined that all was excreted: that it appeared probable that some part was decomposed and appropriated.

Prof. E. T. Darby said that he could recall many instances where there was a hunger and craving for inorganic substances, and where the inference was that they were appropriated. He had known of a child with rickets, which had such an unappeasable appetite for lime that it would crawl to the wall and pick the plaster off till it had eaten all the plaster off a space two feet by three. He had suggested the administration of lime in more suitable form. When lacto-phosphate of lime and cod-liver oil had been given, the child stopped eating the plaster. In the same way when the school.girl eats slate-pencil and chalk, it is because there is a craving for those elements in the system. Thus when pregnant women have similar cravings, it is not fancy; it is a demand of the system which is satisfied when the proper element is supplied. The continuous administration of these elements for months, and perhaps years, may be necessary to produce results. Until this is done, we cannot say that their administration has no effect. It may not always show in one child, or in one generation. Dr. Darby quoted the anecdote of Agassiz, the great naturalist, who advanced the theory of fish-eating for brain-development. Meeting on the sea-shore a fisherman's child which had been raised on fish-diet, but had a very small head and deficient brain, it was pointed out to him as disproving his theory. He quietly replied: " But what would that boy have been if he had not eaten fish?"

And so with the teeth. There is no telling what the result might be if the lime-salts were administered faithfully and continuously. He believed it would work great results.

Dr. Atkinson said that he was happy in the fact that the Association had come to its right mind, and begun to investigate principles. The great difficulty lay in calling the same thing by different names, and different things by the same name. What is the function of iron in the system? It is not merely for coloring matter in the blood. The oxide of iron is a constituent of the blood; a slight increase of oxygen gives the sesqui-oxide of iron. The magnet inviting oxygen to hang about the corpuscles with an oxygenating grip,—courting but not marrying—and then we get the breaking up of the courtship: The red blood corpuscles coming to the air-cells in the lungs, four per cent. of the oxygen is depolarized--non-magnetized--the corpuscles coming back to the lungs to be remagnetized. We must get rid of the stumbling block of *organic and inorganic.*--Subject passed.

### SECTION III. LITERATURE AND NOMENCLATURE.

Dr. Atkinson read a paper entitled "Ripening and Ripeness." This paper was so thoroughly condensed, being almost a succession of aphorisms and definitions, that an abstract fails to convey any clear conception of its depth and wisdom. He said:

Ripening is the completion of process. Production is the leading forward of something which is led; this involves conscious and subconscious order of movement. Consciousness is independent of time and space until arrest of movement produces sub-consciousness manifiested in the senses.

Sound mental action is regular in accordance with purpose; unsound mental action is irregular and purposeless. Perspicacity of nomenclature demands the settlement of the question of germs, the foresteps in kingdom, class, order, genus, species, and varieties. Mass changes only have been studied. Molecular changes have been ignored. The first requirement is to determine the lowest form of body, capable of storing heredity. If the researches thus far made, are to be relied on, the smallest corpuscles are able by fission, or by producing spores to perpetuate their kind. But little is yet known or definitely settled regarding these foresteps. In attempting to account for the origin of the atom, we become lost in the cloud mass.

Atoms are unoriginated eternities. We have to begin with an assumption or postulate as a base, given this starting point, and we are led on to ether, gas, vapor, water, colloid and solid. Assume the atom and we reach molecules, corpuscles, tissues, organs and systems.

When the molecules are arranged in regular order the corpuscle is produced, further measures of energy fit it for arrangement into tissue. Organs are built of tissues and systems of organs.

Ether is the diffusion of atomic mass, disturbance in the tension of ether generates molecular mass.

Air supports combustion, water puts it out, and the earth is formed by precipitation of the ash and dissipation of the gas.

Thus, the earth, the atmosphere, and the empyrean are made to appear as the observable sphere of functional activities.

Dr. Kulp, Davenport, Iowa, read a paper on Nomenclature. He said that in all science the nomenclature was of the first importance. Each term should convey a definite idea. In dentistry there is only a general classification, each writer has his own terms. The profession demands a uniform nomenclature.

The following is a general outline of the system of nomenclature for the teeth and their surfaces, proposed by Dr. Kulp, which has been successfully used by him in his classes.

The surfaces of the six anterior teeth, upper and lower are, labial, lingual, proximal and occluding.

Of the bicuspids and molars, buccal, lingual, proximal and occluding.

The longitudinal surfaces (applicable to all the teeth) grinding, middle and cervical thirds (from cutting edges to alveolar process.)

To individualize the proximal cavities, those of the central incisors are—centro-proximal and latero-proximal.

Of the right and left laterals, centro-proximal and cuspo-proximal.

Of the cuspids, latero-proximal and bicuspo-proximal.

The first bicuspids—cuspo-proximal and bicuspo-proximal, buccal cusp and lingual cusp.

The second bicuspids—bicuspo-proximal, molo-proximal, bicuspo fissure, buccal cusp and lingual cusp.

The first upper molars--bicuspo-proximal, molo-proximal, anterior fissure, crown fissure, posterior-buccal prominence, anterior buccal prominence, lingual prominence (the upper molars being tri-cuspid as a rule.)

This system being carried out to its fullest extent, every possible cavity, or any portion of each and every tooth, upper and lower, is clearly and definitely located, in a single term, terse and simple.

Papers and subject open to discussion.

Dr. Foster, Baltimore, in the chair *pro tem.*

Dr. C. W. Spalding said that the most severely felt want in the profession was the exact definition of terms, especially in the manipulative and mechanical portions of work. In the system here proposed, the terms are as brief and definite as possible, but we need a system of nomenculture which shall extend beyond the mere crown of a tooth and beyond operative dentistry. As it is, all our terms are confused, the same word often expresses very different ideas on the one hand, while different words express the same idea on the other.

Dr. Thompson, Topeka, enquired why the terms, "mesial" and "distal" brief and plain terms, now generally used, were rejected.

Dr. Kulp. Because the words "mesial" and "distal" convey either no meaning or a false one to him who has only an ordinary primary education, as is too often the case with our students, while a term derived from the names of the teeth involved defines itself. The term distal, implying most distant, conveys a false impression when applied to a cavity of the anterior teeth, indicating the palatal surface.

Dr. Taft. The subject of nomenclature is of the greatest interest and importance to our profession. Accuracy in conveying thought depends upon the words used; they should be definite and accurate; easily and readily apprehended. The use of language in the expression of thoughts is modified as the profession grows in knowledge of the language of science. Everything possible should be done for the attainment of the highest point. Discussions in associated bodies tend to the attainment of definite results. Teachers should lead in the right path. The effort of Dr. Kulp looks to the teachers in our profession; any system generally adopted by them will come into general use in the future. The same system must be adopted both in our Institutions of learning and by those who write for the profession. The language used is often inappropriate, or conveys no definite idea. The plan proposed by Dr. Kulp is more definite and more universally applicable than anything heretofore proposed. It is sometimes desirable to use different words to avoid tautology. Masticating would be more definite than occluding. Occluding signifies "to come in contact," but all surfaces do not come in contact.

Dr. W. N. Morrison, St. Louis. Seven or eight years ago, Drs. Judd and Dean produced a monograph on this subject, which was adopted in Chicago and St. Louis. The terms mesial and distal when once defined and learned, looking to and looking from the median line, are simple and clear. Dr. Kulp's system is more complicated.

Dr. Taft. I am quite in favor of the terms used in the paper. Proximal is more easily understood; we should never go far beyond the apprehension of those who listen.

Dr. Morgan. The term occluding strikes me as incorrect when applied to the front teeth. When they are at rest they do not occlude. Cutting edges are not occluding surfaces.

Dr. C. W. Spalding. These points were very carefully discussed in the section. The terms grinding, masticating, and incising are each applicable in its own place, but occluding is more universally applicable; it is the only term that can be made to answer for all; it avoids multiplicity of terms and is liable to fewer objections; masticating applies to some, incising to others, occluding will answer for all. Distal and mesial have no particular advantage; they are not plain English, and not understood by patients. Cuspo-lateral is plainer than disto-lateral. Mesial applies to surfaces looking to the centre; distal means more remote, but they are arbitrary in their application.

Dr. Atkinson. Wherever we attempt to name, we must take nature's standard. We must avoid all complications. Occlude is the term *par excellence* to stand singly, signifying simply "bringing together." The terms we now use are picked up higgley-piggley; an *omnium gatherum* of all time. Some day we will print a primer which will make a tip-top blue blossom of a dentist in three months.

Dr. Taft. In this comparison between the terms, occlusion is the incipient step in mastication.

Dr. G. J Friedrichs, New Orleans. At every meeting of the Association the last system of nomenclature offered is considered the best. I see no outcome; at Niagara our report was almost snubbed. There is only one way of arriving at a uniform basis. Let a committee, either of College Faculties, or from this Association, revise by sections this or some similar paper offered by the section, and then let it be adopted. Otherwise it is all balderdash.

Dr. Sitherwood, Bloomington, Ill., said that it was a matter of necessity that whatever system was adopted should be one readily comprehensible by both patients and students.

Dr. Allport considered the paper open to criticism. The molars were grinding teeth; the cutting edge of the incisors was not an occluding surface. Different terms must be used if we would have accuracy.

### SECTION IV. OPERATIVE DENTISTRY.

The acting chairman, Dr. Darby, announced one paper, The Painless Operation, by Dr. J. A. Robinson, Jackson, Mich., and recommended several subjects for discussion: viz.

| | |
|---|---|
| Bridgework: discussion to be opened by | Dr. E. Parmly Brown. |
| The Herbst Method " " " | Dr. M. L. Rhein. |
| The Perry Separators and Matrices " | Dr. Darby. |
| Half-bicuspid crowns, " | Dr. Noble. |
| Regulating Exhibition, " | Dr. Southwell. |

Dr. W. C. Barrett read Dr. Robinson's paper, prefacing it by saying that "Uncle Jerry," as he was affectionately styled by all who knew him well being unable to attend, had submitted his paper to the Section, which had accepted it, and designated him as the reader. Having had no opportunity to familarize himself with the manuscript, he feared he might fail to do it justice. The following is a brief summary of the paper:

There is a universal desire to relieve or prevent pain. Tooth extraction is not desirable; it is not a blessing to the patient. We must be conservative rather than destructive. We encounter the greatest difficulty with friable teeth with anæmic conditions; palliative treatment becomes a necessity; an experience of fifty years has overcome many obstacles. The use of Carbolized Potash—Robinson's remedy—affords the most effectual relief. The brief pain occasioned by the application is nothing compared to that of extracting, and we can give positive assurance that the latter will not hurt. If the nerve is exposed first apply clear carbolic acid to cauterize the nerve, then apply the remedy, and let it remain ten minutes; open with large burr—the excavating will be painless. If there is any soreness give the tooth rest; prevent occlusion with antagonizing teeth which would keep up irritation—shorten the cusps with corundum wheels. Gold is the best material for filling; amalgam is too often a failure; the textile foil prevents all shock from thermal changes. It has been said that it does not contain sufficient welding properties to hold a cap of gold, but I have never had a single failure with No. 6 foil welded to textile foil. Amalgam will be used for many years to come. Sinners are sometimes made better by good company; so of amalgam with textile foil; it makes a putty like mass, composed of silver, tin, platinum and gold. We must toil till we see the salvation of tooth sinners; then like Simeon of old, we can "depart in peace."

Dr. E. Parmley Brown, Flushing, L. I., opened the subject of Bridgework. He said that as many dentists had never seen any of this new work he would only say that it was worn at least two thousand years ago, a genuine piece of bridgework, dug up in Etruria, had doubtless been the property of an ancient princess; that she, in laughing would have shown a gold band both inside and out, a complete "give away;" that recent improvements in dentistry had done away with the gold band, having however an unsightly gap between the teeth and the gums. As another step, far in advance, he now offered bridgework (of which he exhibited specimens) with no metal visible inside or out, and no spaces for food to lodge, but a close fit all around—sweet and clean and serviceable. He described a case where the right upper molar was healthy but pulpless—a mere shell suitable for covering; the second bicuspid ulcerated and useless; the first bicuspid all right. He extracted the festered root and put the new tooth right up into the wound, the porcelain tooth apparently growing out of the gum. The patient was so well pleased that he wanted to be "done so some more" for the other side of the mouth.

Dr. Thomas, of Detroit, inquired how the strain on the teeth of attachment was avoided?

Dr. Brown replied that there was no difference in that regard between his and any other method.

Dr. M. L. Rhein, New York, opened the subject of The Herbst Method (which was left unfinished at Saratoga.) He said that during the past year he had modified his method somewhat; that he does not now complete his work by the Herbst Method.

The special advantages of this method are its superiority in adaptability of gold to the walls of the cavity, without retaining pits or undercuts; the avoiding of nervous shock to the patient of malletting, and the great saving of time over any other method. The larger and more extensive the approximal cavities in molars and bicuspids, the easier and more quickly in proportion they can be filled by the Herbst Method, in comparison with other methods. The only disadvantage is that the gold is not as thoroughly condensed, not as much impacted as with the mallet; therefore he has modified his method, and finished the last one-third of the work with the electric mallet, thus obtaining contour, and thoroughly condensed masticating surface. Dr. Böedecker has done the same. Insert three-fourths, or seven-eighths of the filling, up to the coronal surface, with the Herbst Method, then compact with the mallet and get all the density required.

Dr. Herbst himself does not acknowledge that he cannot impact as much gold as by any other method, but others have not been able to do it. Cavities must all be reduced to simple cavities, applying the matrix for the missing wall in approximal cavities; a thin steel matrix held in place by heated shellac, making the most difficult approximal cavities as simple as crown cavities in molars.

Dr. Rhein exhibited specimens of work done by Dr. Herbst; a lateral in which two-thirds of the crown was built up in forty minutes. Crown cavities in molars, the entire operation finished in from six to fifteen minutes, etc. Some of the work was done with tin foil. Some of the teeth were split to show the perfect adaptation of the gold to the walls of the cavity. A perfectly pure very soft gold is prepared especially for this method, and used without annealing. The original is made by Wolrab in Germany. S. S. White and Williams now offer American imitations, but which do not work like the original.

Dr. Allport. Does Dr. Herbst claim to weld tin?

Dr. Rhein. The specimens have that appearance. The Wolrab gold works like rubbing the burnisher in a roll of butter.

Dr. Darby exhibited Perry's Separator, also various forms of matrices, and appliances for holding the matrix in place. He said that Dr. Perry was one of the first to follow Dr. Jones in his method; the new separators are more out of the way, and control the pressure more evenly. He exhibited one adjusted to the incisors, separating the central from the lateral, with the matrix in position on the distal surface. Dr. Jack's method held the matrix in place with an orange wood wedge. The present instrument is on the principle of that used for measuring the foot, or the monkey-wrench, binding the matrix between the teeth. For separating molars and bicuspids it fits the teeth as the saddle does the horse. They do not interfere with the dam, do not incommode the patient, and are out of the way of the operator. Dr. Darby also exhibited Dr. Woodward's matrix, made of steel, phosphor-bronze, brass, or copper. If of steel, the Seth Thomas watch spring is good, cut with plate shears. It is made with a lobe bent back upon the masticating surface of the adjoining tooth, which prevents it from slipping up to the gum. When the space is very narrow a wedge can be used shaped like a knife blade; it is less liable to break than a wooden wedge, and holds the matrix in place while holding the teeth a little apart. Phosphor-bronze is pliable and malleable and readily adapted to the space, forming a matrix holder and separator combined.

Dr. Noble, Washington, exhibited the half-bicuspid porcelain crown; Howe crown, or tooth, with four pins, and screw in root. This modification is adapted to the outer half of a bicuspid tooth, supplying an outer cusp to the natural inner cusp and root, and doing away with the labor of building up. It is faced with amalgam on cement filling, with a gold band embracing the cervical margin, and makes a strong and useful operation. Dr. Noble also showed Dr. Donalson's most recent invention; a machine for cutting barbs on all sides of his broaches, but not in a circle.

Dr. Southwell, of Milwaukee, exhibited an original invention for throwing the breath exhaled by patients away from the operator; a curved plate resting on the upper lip, to which is adjusted a shield which receives the air from the nostrils and throws it up and off to the right or the left, and away from the operator, especially for operating on the upper teeth of a catarrhal patient. Dr. Southwell also read a paper entitled "The Health of the Dentist" as affected by the close relation with patients, especially in the matter of inhaling the hot, moist breath, loaded with noxious exhalations of the lungs and nasal passages. He said that as a man cannot exist more than two minutes without air, while he may live eight or ten days without food, the necessity for air in proportion to food was as four to three thousand, and yet how much more we think of the food we eat than of the air we breathe! The oxygen retained is equal to the carbonic acid gas exhaled; a slight percentage of impurity may be tolerated.

In inserting large fillings we are frequently so placed below and in front of the patient that the hot breath is directly within range of our lungs; we become light-headed and dizzy from inhaling carbonic acid gas, and nervous exhaustion results. In addition to this there is the constrained position, imperfect aeration of blood, vitiated atmosphere, underfeeding, etc. These things should have our earnest consideration, especially the importance of ventilation. With this little apparatus one fruitful source of ill health will be obviated.

Dr. A. E. Mattison, of Chicago, exhibited an appliance which had been successfully used on a boy twelve years of age, who in playing had accidently fallen and knocked out the central and lateral incisors. The process was broken off, hinging at a point near the foramen of the teeth; the teeth were knocked entirely out and picked up from the ground. The root canals were dressed with eucalyptus and filled with gutta-percha. It was found very difficult to force them up into place. With the splint shown, which was cemented to the adjoining teeth, they were retained in place. The teeth were easily kept clean and sweet, and the boy never lost a meal. It proved simple and effective. He was assisted in the operation by Dr. Frank Gardiner. Dr. Mattison also exhibited his improved crowns. These are perfectly contoured shells, made of gold, lined with platina, giving the greatest amount of strength with least amount of material. They are readily thickened where required, as on the masticating surface, without melting the shell which can be used as a crucible, melting the solder or gold scraps within the crown, thus securing correct articulation with the occluding teeth. Another great advantage is the ease with which a porcelain face can be inserted, by cutting out the face of the shell, leaving a narrow band at the cervical margin, and inserting a platinum ring the width of the band, which rests on the end of the root, forming a shoulder, which is soldered to the shell. For the porcelain front use a plain tooth, either with the pins, or by grinding a dove-tailed slot, giving room for the screw-pin, which is inserted in the root and filled in with amalgam. The pin is of platinum wire, eighteen or twenty gauge, with screw-threads, the upper end bent so as to fall into the slot of the porcelain front when that is inserted. The crown is anchored to the root with amalgam, and the front cemented to the crown with oxy-phosphate. The root can be perfectly filled through the opening in the crown before the porcelain face is cemented on. A plaster cast of the adjoining teeth, and a cast of their antagonists, secures a perfect adaptation and articulation. The root must be in healthy condition and the nerve-canal filled at the apex. The end is to be ground off below the margin of the gum in front, and within an eighth of an inch of the gum on the lingual surface. The crowns in assorted sizes, as also the gold and platina plate, and the dies and counter dies for making the shells can be obtained from the S. S. White Manufacturing Co.

Wednesday afternoon was devoted to Clinics, with Drs. Gardiner, of Chicago, Field, of Detroit, and Smith, of Minneapolis, as committee.

Dr. E. Noyes, of Chicago, made a handsome contour filling, in a central incisor, using cohesive foil and steel mallet.

Dr. E. Parmley Brown, using the electric mallet, made four very large contour fillings in the proximal and grinding surfaces of the first and second left superior bicuspids and first molar, with his usual grace and thoroughness.

Dr. T. T. Moore, of Columbia, S. C., made a very artistic contour filling in the anterior and palatine surfaces of the first superior right molar; also using the electric mallet.

Dr. I. C. St. John, of Minneapolis, made gold contour filling in a proximal cavity of a central incisor, using a wooden mallet.

Dr. C. A. Timnel, of Hoboken, N. J., made a large compound filling in a first left superior molar. He illustrated the Herbst method. The filling was dense and solid but the proximal surface was not contoured, it having been previously filed flat.

Dr. M. L. Rhein, of N. Y., made a large compound filling in the posterior proximal and grinding surfaces of the left superior first bicuspid. He used Wolrab's German gold, by the Herbst method, for about two-thirds of the operation, lining the walls of the cavity to the margins and slightly annealing the gold and using the mallet to contour. The tooth was built up in good shape.

Dr. A. W. Harlan, of Chicago, treated two cases of Pyorrhea Alveolaris, using Dr. Allport's new spring scalers.

Dr. J. R. Patrick, of Belleville, Ill., exhibited his device for striking up gold crowns, and Dr. Swasey, of Chicago, showed his apparatus for making corrundum points, wheels, etc.

Dr. Mattison, of Chicago, inserted one of his porcelain front, gold and platinum crowns on a left superior lateral incisor root. The tooth presents a very natural appearance and the operation promises to be a success.

The clinics were watched with a great deal of interest, and many new points were given.

WEDNESDAY, 8 P. M.

The President, J. N. Crause in the chair.

SECTION V. ANATOMY, HISTOLOGY AND MICROSCOPY.

Dr. Frank Abbott, N. Y., read a thoroughly scientific paper, entitled "Studies of the Pathology of Enamel of the Human Teeth, with special reference to the Etiology of Caries."

Dr Abbott first described the Micro-steriopticon of Stricker, of

Vienna, an instrument by which the field of an ordinary slide, magnified 12,000 diameters, is projected upon a screen, so that a thousand persons can study it at once. Theories regarding tooth structure are thus proved or disproved. Dr. Abbott said that he expected to receive one of these instruments in a few weeks for the further prosecution of his researches in this direction. He also said that he was indebted to his friend and teacher, Carl Heitzman, for the very fine micro-photographic illustrations of his present paper, by which he was able to prove the truth of the positions taken.

He spoke of the very great importance of the study of the Pathology and Etiology of caries. That there was a marked difference, not only in individuals, but also in races, in the liability of teeth to decay. That civilization had been accused of being a prominent factor of causation, but that this would not explain the rapidly growing tendency towards this disease, under all circumstances; that there must be an anatomic sub-stratum, aside from acquired or local causes, the latter being mere auxiliary agents, fostering more remote causes. He had examined a large number of specimens, ground into thin slabs with the soft parts, their living matter carefully preserved, the results being highly satisfactory in explaining the tendency to decay as due to deficiency in anatomical structure. Before entering into the description of anomalies, he described the structure of normal enamel and its relations to dentine, as discovered by Bödecker; the connection between the dentinal and enamel fibres, the distribution of living matter in tooth substance, even in the interstices separating the minute sub-divisions of the enamel rods, thus raising the enamel to the dignity of living tissue instead of a mere deposit of calcareous elements; that the dentinal canaliculi upon entering the enamel often became enlarged in pear-shaped cavities containing protoplasm, which was in direct connection with the termination of the dentinal fibres, and on the periphery, with the fibres of living matter of the enamel, these enlargements being more regular in the teeth of young persons.

The first plate represented a temporary molar, with a protrusion of dentine into the enamel, with a fluted surface, the centre of the protrusion occupied by protoplasmic formation, the enamel nearest the protrusion destitute of prisms, the zone above the protrusion but slightly brownish while the prisms of the enamel exhibited a very distinct brown pigmentation.

The second plate represented a permanent canine, with a pear-shaped or conical protruberance, without distinct boundary, on the buccal surface, occupying nearly one-half the enamel, the adjacent enamel showing indistinct rods, but occupied by brownish globular fields resembling the interglobular spaces of dentine. The rods were slightly pigmented rods, more wavy than normal. There were no interglobular spaces in the dentine of this tooth.

The third plate was a diagram showing the stratification of enamel, layers varying in width, the broadest portion always corresponding to the cusps, the narrowest to the neck of the tooth, the enamel rods of the cusp-layers invariably vertical to the surface, while those of the neck-layers may be parallel to it, this construction sometimes varying at the outer periphery of the enamel. Higher powers of the microscope show that the lines of stratification do not alter the general course of the enamel prisms, an exception occurring only at the peripheral portions of enamel occupied by the tapering ends of the neck-layers which may exhibit enamel rods almost parallel with the surface of the enamel, while at the outer periphery of the cusp-layers the enamel rods would invariably be more or less vertical.

The stratification of the enamel is of the utmost importance for the understanding of pigmentation and granulation, these both corresponding with the general strata of the enamel, showing in a longitudinal section a fan-like appearance. The stratification of this tissue bears close relation to the history of its development. The first enamel cap of temporary teeth is broadest in the direction of the cusp, and tapering towards the future neck, subsequent layers forming on the plan of the first. Illness of the mother before delivery, or of the infant after delivery, might cause an interruption in its construction, leading to stratification, pigmentation and granulation, these conditions invariably involving a deficient deposition of lime salts.

Fig four illustrated the irregular course of pigmented enamel rods. In normal enamel longitudinal sections exhibit slightly wavy rods, interlaced by small bundles in a transverse direction, the curvature becoming gradually less, till it is nearly straight, close to the surface. Where there are deviations from the rule, they lose all regularity, oblique and longitudinal blending as in the graining of lignum-vitæ. In such cases the curvature of the rods may remain marked up to the surface, showing groups of transverse sections at the outer surface, perhaps on one portion only, the rest of the enamel being normal. With this curly appearance pigmentation is combined, and the interstices between the rods are wider than normal, all indicating deficient calcification. The dentine is also freely supplied with interglobular spaces, another sign of deficient calcification. The friability of such enamel is so great that it is extremely difficult to obtain an unbroken slab. With low powers of the microscope the broken ends of the enamel rods look as if corroded. The enamel rods are unusually narrow, the interstices correspondingly wide, and their tenants—the enamel fibres—very prominent; the reticulum near the intro-zonal layer is also very prominent.

These conditions may occur both in temporary and in permanent teeth, and may be combined with pigmentation.

Another result of deficient calcification is seen in the congenital white or yellow spots, which if broken into are found of the consistency of chalk. This is mis-called "white decay," but is a congenital defect due to deficient calcification, and is closely allied to the rows of pits with no enamel in the bottom of the depressions. These are also congenital and due to the same cause. Some portions or all of the crown may be covered by a brown or yellow substance which is not enamel, but which is soft and easily removed, leaving the dentine exposed and extremely sensitive. These might all be termed exaggerated cases of pigmentation. The form which is most common, presents to the naked eye only a slight yellow-brown discoloration. The microscope shows that the pigmentation occurred during the formation of the structure. If in non-stratified enamel there is no demarcation of the brown spots, but faintly marked offshoots taper off towards the dentine. In stratified enamel the deepest stain corresponds to the boundary of the strata, tapering towards the neck, fading towards the proximal line, showing a pan-like configuration in longitudinal sections. This pigmentation may invade all the layers of enamel, being perhaps more marked in the deepest portions. The microscope shows that the brown discoloration concerns the basis-substance of the enamel rods only; that the widened intestices are due to deficiency in formation, and not merely to contrast in color; that the transverse lines are also enlarged; that the enamel fibres are more conspicuous than even in normal enamel of temporary teeth. Pigmented enamel also readily stains with an ammoniacal solution of carmine. It is not yet known what disturbance of the enamel organ, interfering with the proper construction of the basis-substance and the deposition of lime-salts, causes the brown discoloration. All pigments of the body depend upon the coloring matter of the blood, but careful studies in the history of the development of enamel must be made before the solution of this question can be reached. One point is positive—viz. that pigmentation is congenital, involving both permanent and temporary teeth. Acquired pigmentation being rare except as the result of caries. Caries invading spots congenitally pigmented causes an orange discoloration, which readily takes the carmine stain, the congenital brown, acquired orange and artificial red, mingling in beautiful shadings.

The granulation of enamel is a peculiar pathological condition by no means rare, and consists of pear, spindle and club-shaped spaces in the middle of the substance of the enamel, as well as at the junction of the dentine and enamel as heretofore recognized. These spaces are enlargements of the interstics between the prisms and the tenants of the interstics. Plate VI represented this condition under high powers of the microscope.

The interprismatic spaces of the enamel bear some resemblance to the interglobular spaces of the dentine, both conditions being present in highly marked degree in one specimen. The deficiency of basis-substance and lessened amount of lime salts renders granular enamel both brittle and prone to decay. Stratification, pigmentation, and granulation are all pathological conditions of the enamel meaning a deficient formation of the basis-substance and also decreased deposition of lime-salts, and are of the highest importance in the etiology of caries.

The conclusion reached was that ailments of either the mother during gestation, or of the infant during the earliest periods of life, cause these anomalies; that they are more common in high life than among hard working people, persons of refined habits, debilitated by the luxuries of civilization. The pathology of enamel has no literature. In the discussion which followed:

Dr. Pierce, of Philadelphia, said that he wished to congratulate the Association on the opportunity they had enjoyed through this paper, of examining diagrams illustrating vital conditions, such as were not often offered to any society. He desired to call attention to the fact that No. 1 illustrated conditions that must have been confirmed in utero. Between the dentine and enamel the line of first calcification must have taken its shape as early as the fifteenth or sixteenth week of embryonic life. It must have had its peculiar form at that time, before being rendered permanent by the deposition of salts of lime. This afforded a valuable lesson in the vitality of structure, and how readily it is modified by lack of nutrition. The abnormal projection of dentine structure was doubtless due to some peculiarity of nutrition currents at that early period. It teaches the readiness with which tooth-structure is modified by systemic conditions.

Diagram No. 3 afforded the same index as the concentric lines of the tree periods of growth. In the enamel of the diagram referred to there were clearly indicated periods of growth and arrest—nutrition and cessation—affording another proof of the liability of the teeth to be influenced by systemic conditions. And so on through all the diagrams, each representing a special phase, but all teaching the same lesson—the undoubted modification of these hard structures by systemic conditions.

Dr. Atkinson said that he was delighted with the specimens of proof of idealism in observers in histology of tooth-structure; anomalous cases were taken as the regular order; that there was much assumption, much unwarranted deduction and misuse of terms. The enamel pulp is not always systematically developed into enamel rods and prisms and spaces; rods are produced when required as protection. The significance of the enamel caps is not understood. What becomes of the enamel cap of the embryo is not known. Is it worn off? And then we have to consider the many varieties of teeth in the lower orders of animal life; the teeth of the carnivora compared with those of the inhabitants of water, etc. The fact of nutrition in enamel settles the question whether it is mineral, vegetable or animal. The first diagram shows nothing but a rift in the enamel cap, allowing the amelo-blasts to press beyond the normal line. It is not so much the want of lime as lack of pro-rata; a difference in the consolidation of lime garnules. What that is is too fine to be presented till after many more observations. He said that he hailed the ambition of any one to work in this direction; no matter what the object was so that the work was done; that he was glad to see any statements made, even though erroneous, that would bring our attention to these studies. What we call decay is unbuilding. A man with a microscope sees a keyhole, but he turns around to where there is no door—goes back to the old ideas. It is in vain to have the microscope without the trained brain to interpret for the trained eye. We owe a great debt to Bördecker for his investigations, and thanks to grand Carl Heitzman. It is of very little use to diagnose a case if we have no remedy. We know antecedent and sequent; we don't know cause. What is sterilized earth? It was said we must have mineral before vegetable—vegetable before animal; the first form of all animal life is the germ. It was said that all diseases must have a microbe to work changes in the body—to ferment all tissues up to the requisite condition. Where the microbe is necessary to make food ferment to produce pabulum is the field for our investigation. We must get rid of the old incubus, organic and inorganic; there is no inorganic—there are two distinct opposites, we must have something to blend them and make them verities.

Dr. Thompson, Topeka, desired to ask Dr. Abbott to say something further about the white spots on enamel.

Dr. Abbott. The only evidence we have of the diminution of lime salts is the greater amount of organic material it must be that much short of inorganic elements. The lime salts are loose, can be scraped off with the thumb nail. The yellow spots look like enamel, but are not; the interprismatic spaces are two or three times larger than normal; the lime salts must be less; the relative proportion is changed. There is a certain amount of satisfaction in having a knowledge of disease even if we cannot cure. With reference to "sterilized earth"—what I said was a quotation from the French, and not cited as authority.

Dr. Brophy, Chicago.—Dr. Abbott said that all defects previous to eruption were congenital. According to some observers, as Prof. Eames, of St. Louis, endorsed by Prof. Black, some defects are the result of absorption, similar to that occuring in the roots of diciduous teeth; due to the same agent as that which absorbs the roots. Such an absorption may take place; has seen the destruction of the enamel of incisors from this cause.

Dr. Abbott. The condition I referred to is, in my judgment, impossible after eruption; the very dark, porous, granular condition of a line running out upon the surface. It is no stretch of imagination that something might flow in and affect all along that line. It may be possible to show that it is a case of absorption; cannot see how it could occur similarly to the absorption of the root.

Dr. Keith, St. Louis, said that he had had a peculiar case in practice. He had a regulating plate in the mouth of a little girl, which covered one of the deciduous teeth, the one to be replaced by the first bicuspid. She was absent two or three weeks. When he removed the plate, he found the crown of the deciduous tooth imbedded in the vulcanite plate, with every particle of the dentine dissolved out; it seemed as if cells had grown up above the margin of the gum and eaten out the dentine, without touching the thin margin of enamel.

Dr. Atkinson. The dentine must be reduced to fluidity before it can be taken up by absorbents. You say that the carneous body does the work! No more than the liver secretes liver. Dr. Abbott gave me an adult tooth that had lost two-thirds of the length of the root; it was purforated as if by drill-holes. He had not had time to cut it yet, but intended to do so. It was a case of retrogressive metamorphosis, but we can not understand that. We are getting hungry for the truth ; we want to pin our ears back and go for it!

Dr. Ingersoll read his paper, entitled Alveolar Dental Membrane. Unity or Duality : which?

Handing his specimens to be passed around and examined, he said that he had no doubt of his ability to read his paper while the specimens were being examined, but was not so certain of being heard or understood.

The following is a summary of his argument: He said that the Dental Membrane offered a subject which commended itself to our consideration; that upon the condition of this membrane depended the health, safety, and comfort of the teeth. Unless it was in a healthy condition the teeth might be filled, and the pulp saved, in vain ; but if the membrane was vigorous the crown might decay, and the pulp die, and yet a serviceable tooth be built upon the root. If the membrane fails, the whole structure is gone. Much study is given to alveolar abscess, to pyorrhea, but the histology of the membrane, as a dental tissue, is scarcely referred to. The enamel, the dentine, the cementum, the pulps —three hard and one soft tissue, springing from the dental follicle, are each fully treated of, but the membrane has passing notice. It is a subject which is full of difficulties. Does it appertain to the dental follicle? Early anatomists regarded the tooth as bone, and the alveolar periosteum as an osseous membrane. In the development of the tooth the crown is first found, then the neck, then the root. The follicular neck is a point of particular interest. (Quotations from Legros, Magitot, Tomes and other authors, on this point.)

The alveolar walls develop simultaneously with the root. Proceeding from the base of the follicle is a line of cells, the formative point of cementum, and another line—the formative organ of the alveolar walls. One develops down to form the socket; the other forms the cementum, the two layers being contiguous. The question before us is, is the tissue known indifferently as dental periosteum, or as alveolar membrane, single or dual? The earliest writers say nothing on the subject. Later authors dismiss it with a few words, speaking of it as a connective tissue, well supplied with blood vessels, investing the root and lining the socket. Different names are given to it, showing varying opinions, but it cannot be studied from authors, they are so shy of mentioning it. Where a mucous membrane rests upon bone, it is intimately connected with it. Analogy would lead us to expect that a true alveola-dental membrane would be in two layers; demonstration proves it. When a tooth is extracted the root is covered with a membrane and the socket is lined with membrane. If the membrane exists in two distinct layers, have they the same origin?

The alveolar lining must be referred to the osseous system. The organ which forms the cementum cannot be the same as that which forms the alveolar walls. They are intimately united, but not one; they are distinct and separable; two layers, of distinct origin and performing different offices, an outer layer of connective tissue, with blood vessels; an inner layer of a fine network of cells, there being a marked difference in their histological character, the fiberous elements of the outer passing insensibly into the network of the inner—the peridentium of the tooth, the periosteum of the bone. However close in juxtaposition they are widely apart in function. Tomes says that two membranes may be assumed, but would be extremely difficult to demonstrate; that the fibrous elements of the outer portion blend so insensibly into the bands of network of the inner part that neither sight nor touch nor any other sense can show any distinction, leaving only a mere inference that it is two rather the one. Dr. Ingersoll said that while investigating this subject he visited Dr. Black to see if he had any specimens that would throw light on the subject. He found one specimen that was perfect in all its parts, and in which the osseous membrane and the cemental membrane appeared entirely distinct. First there was a fine network in the meshes of the cemento-blasts; then bundles of fibres in flattened bands, side by side, passing in oblique and curved lines towards the bone. The reticulated osteo-blasts were much larger than the cemento-blasts. None of the straight lines of the peridental membranes were found in the alveolar membrane. There are thus two membranes, with special functions. The outermost membrane forming the bone, the innermost the cementum. They are similar, but not the same. Tomes says, the development of bone might, with some modification, be applied to the development of cementum. Have they different sources of mineral and vascular supply? If the membrane is dual, two sources would be expected; from the pulp on the one hand from sub-mucous tissues on the other. Pathology will reveal the difference in exostosis and ex-cementosis. Irritation follows the course of the blood vessels. Under the influence of irritants the root membrane takes on abnormal action; this is one cause of ex-ostosis—really ex-cementosis. If the nerves and blood vessels follow the course of the membrane, and it is not dual, why are not both layers affected? Why do we not have both true ex-ostosis or enlargement of the socket, and also ex-cementosis or enlargement of the root, and consequent tooth-anchylosis. When two roots are exostosed the two grow together by impingement, but never the socket and root; hence there is no osseous union. Portions of the socket are removed and inflammation of the membrane induces hypertrophic conditions. Dr. Ingersoll drew the following conclusions:

1. The alveolar and dental membrane are not identical; the root membrane is in separable layers.
2. The two layers have not the same origin, they originate respectively in the osseous system and in the dental follicle.
3. They differ in structure; banded and reticular.
4. They have different functions; one forming the socket, the other the root.
5. They have different sources of vascular and mineral supplies.
6. Pathology points to the same fact; there is no cemented union between the root and the socket.
7. The rapid mineral absorption of the root, without exfoliation, is due to the special nature of the dental membrane as distinct from the membrane of the socket.

Thus surgery, pathology and physiology all point to the same conclusion, the duality of the alveolo-dental membranes.

Subject open for discussion.

On motion, adjourned to Thursday, 9 A. M.

(TO BE CONTINUED.)

